# Predicting Symptom Relief After Reoperation for Suspected Internal Herniation After Laparoscopic Roux-en-Y Gastric Bypass

**DOI:** 10.1007/s11695-018-3404-8

**Published:** 2018-07-18

**Authors:** Leontine H. Wijngaarden, Sophie L. van Veldhuisen, René A. Klaassen, Erwin van der Harst, Charles C. van Rossem, Ahmet Demirkiran, Steve M. M. de Castro, Frederik H. W. Jonker

**Affiliations:** 1 Department of Surgery, Maasstad Hospital, Maasstadweg 21, 3079 DZ Rotterdam, The Netherlands; 2 Department of Surgery, Rode Kruis Hospital, Beverwijk, The Netherlands; 3 Department of Surgery, OLVG, Amsterdam, The Netherlands

**Keywords:** Internal herniation, Laparoscopic Roux-en-Y gastric bypass, Mesenteric defect closure

## Abstract

**Background:**

Internal herniation (IH) is one of the most common long-term complications after laparoscopic Roux-en-Y gastric bypass (LRYGB). Diagnosis of IH may be difficult, and not all patients with suspected IH will have full relief of symptoms after closure of both mesenteric defects.

**Objectives:**

To investigate possible predictive factors for relief of symptoms in patients with suspected IH.

**Methods:**

All patients that underwent reoperation for (suspected) IH after LRYGB from June 2009 to December 2016 were retrospectively evaluated in this multicentre cohort study. Logistic regression analysis was used to identify predictive factors for pain relief after closure of the mesenteric defects.

**Results:**

A total of 193 patients underwent laparoscopy for (suspected) IH during the study period. The median interval between LRYGB and reoperation was 18.3 ± 19.0 months. In 40.2% of cases, IH was identified on computed tomography (CT), and IH was objectified during surgery in 61.1%. Postoperative symptom relief was observed in 146 patients (77.2%). For patients in which IH was present during surgery, 82.8% had relief of pain postoperatively, as compared to 68.5% for those procedures in which no IH was found. The only significant predictor for postoperative pain relief was a swirl sign on CT (OR 4.24, 95%CI 1.63–11.05).

**Conclusions:**

Pain relief after closure of the mesenteric defects for IH remains unpredictable. A positive CT for IH was a predictive factor for symptom relief after reoperation for (suspected) IH after LRYGB. However, many patients benefit from closure of the mesenteric defects, irrespective of perioperative presence of IH.

## Introduction

The laparoscopic Roux-en-Y gastric bypass (LRYGB) has become a common bariatric procedure leading to satisfying long-term results in both weight reduction and reduction or even remission of comorbidities of morbid obesity [1–4]. However, due to altered bowel anatomy after LRYGB, internal herniation (IH) can occur through either the mesenteric defect of Petersen’s space or the mesenteric defect of the jejunojejunostomy (JJ-stomy) during follow-up [5]. The reported incidence of internal herniation varies widely between 1.6 and 9.3% [5–9]. The typical presentation of patients with an internal herniation is intermittent, postprandial, upper abdominal pain, sometimes accompanied by nausea and vomiting [10, 11]. Less frequently acute intestinal obstruction with or without bowel strangulation may occur, in which case emergency surgery is indicated. The mean interval between LRYGB and presentation of IH varies between 15 and 26 months in larger series [12, 13].

The presence of a ‘swirl sign’, caused by rotation of the mesenteric vessels on computed tomography (CT), is the golden standard to diagnose an IH, albeit varying sensitivity outcomes of CT have been reported [14–16]. Typically, management of IH consists of a reoperation with repositioning of the herniating bowel and closure of both mesenteric defects [17, 18].

Since IH may present with non-specific symptoms, preoperative diagnosis may be difficult and negative explorations have been described [8]. In some patients with typical intermittent pain symptoms and a clear ‘swirl sign’ on CT scan, actual visible IH may be absent during surgery. In case of open mesenteric defects without objectified IH, patients may still benefit from closure of the mesenteric defects. Even more strikingly, some asymptomatic patients may have IH clearly visible on abdominal CT or during reoperation but do not benefit from closure. Therefore, outcome of pain and symptom relief after mesenteric defect closure seems to be highly unpredictable in literature.

The aim of this study is to investigate patient-related factors and intraoperative findings in patients with delayed closure of mesenteric defects, in order to predict postoperative symptom relief after reoperation in patients with suspected IH after LRYGB.

## Methods

### Patient Selection

Mesenteric defects were not routinely closed during LRYGB at our institutions until January 2017. Generally, we differentiate between patients readmitted with acute symptoms of IH, possibly with abdominal tenderness and hemodynamic instability, and patients with a more chronic or intermittent presentation. In case of acute symptoms, urgent abdominal CT is typically performed, with subsequent laparoscopy if IH is suspected or if serious abdominal symptoms persist without any diagnosis. Patients with a more chronic or intermittent presentation first undergo treatment with increased dosage of proton-pump inhibitors (PPI) and mucosal protective drugs (MPD). If complaints are persistent, an abdominal CT, gastroscopy (EGD), and/or reoperation are performed for suspected IH. For this analysis, all patients who underwent reoperation after LRYGB for suspected acute or chronic IH from June 2009 until December 2016 at the three bariatric institutions were retrospectively reviewed. Patients were excluded if other abnormalities were found during reoperation that could explain the complaints (e.g., obstructing adhesions) or in case mesenteric defects turned out to be closed during exploration (e.g., by adhesions).

**Fig. 1:**
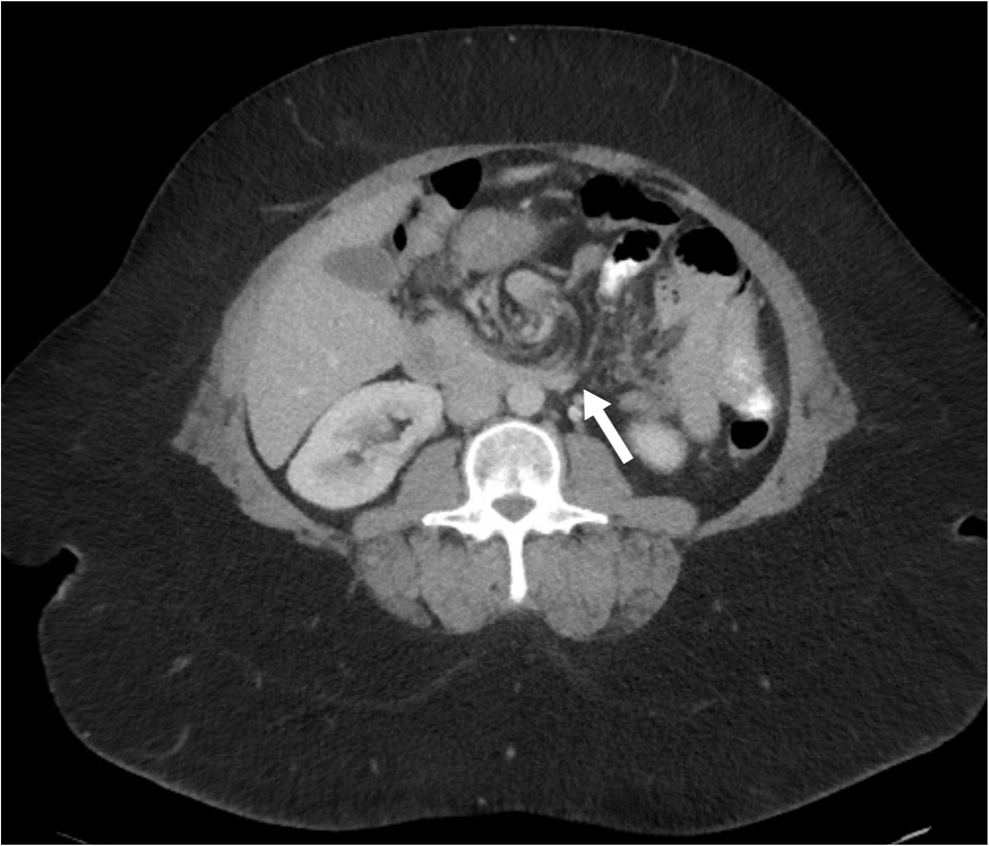
Transverse CT scan through mesentery shows a subtle swirl sign of the mesenteric vessels of a 45-year-old woman with internal herniation

**Fig. 2:**
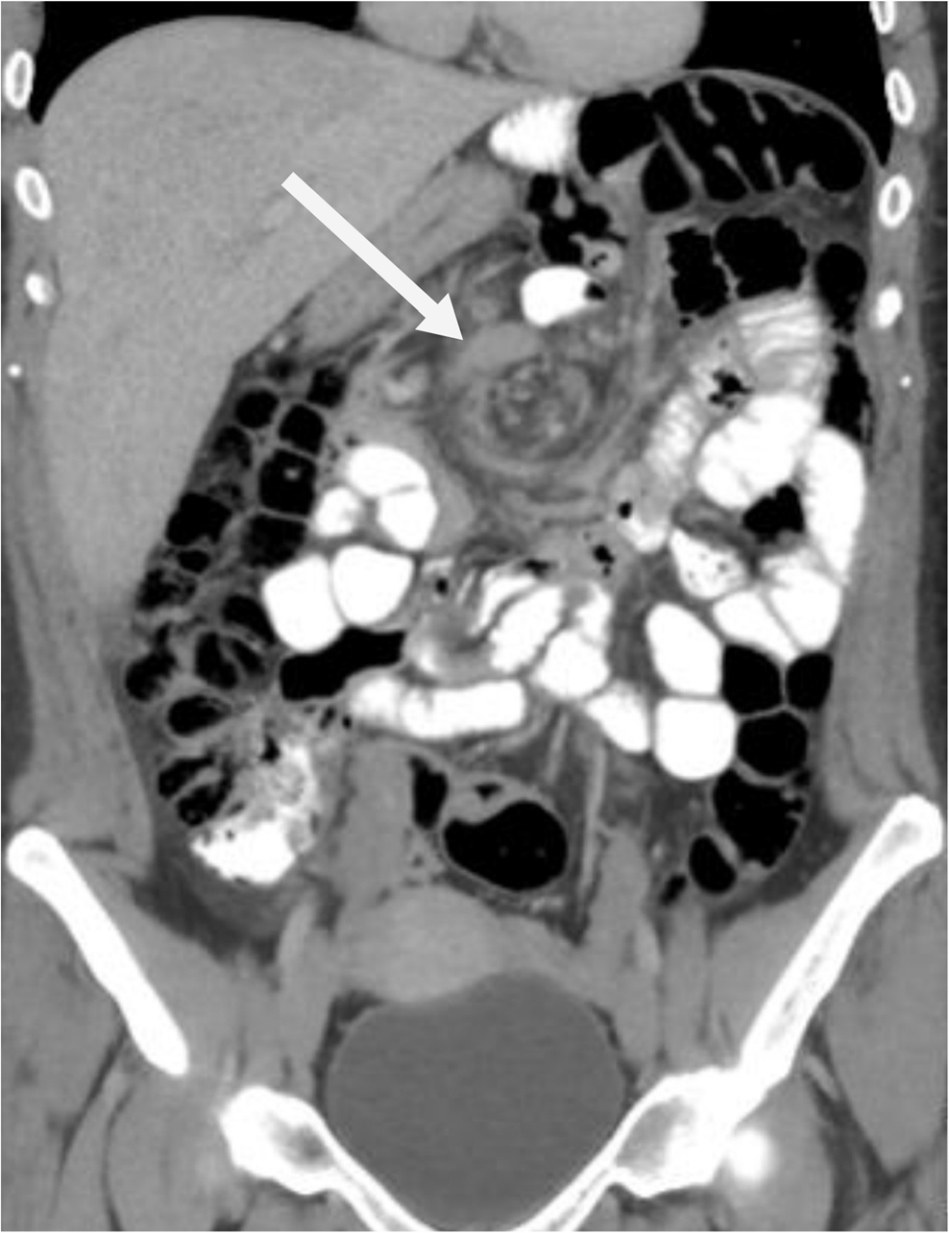
Sagittal CT scan of a 360° swirl sign in the mesenteric vessels of a 34-year-old woman with internal herniation

All CT scans were interpreted both by a radiologist and an experienced bariatric surgeon. We defined a CT scan as positive if a swirl sign with an estimated amount of swirl of at least 180° was seen. Two examples of a positive swirl sign on CT are shown in Figs. 1 and 2. Intraoperative findings were investigated and postoperative pain relief was assessed for all patients. Subsequently, predictors of internal herniation during reoperation and predictors of postoperative pain relief were investigated. Pain relief was scored positive if the patient did not have postprandial, upper abdominal pain 3 months after reoperation.

### Surgical Procedure

In our institutions, LRYGB was performed without primary closure of the mesenteric defects. The LRYGB was performed with an antecolic approach. After division of the jejunum, the biliopancreatic limb was positioned to the left of the alimentary limb while rotating the alimentary limb to the right. The jejunojejunal limb and biliopancreatic limb were positioned to the left of the alimentary limb.

Reoperations were performed by an experienced bariatric surgeon using laparoscopy with inspection of the bowel anatomy of the alimentary limb, the biliopancreatic limb, and the common limb. If internal herniation in Petersen’s space or through the defect of the jejunojejunostomy (JJ-stomy) was present, the bowel was repositioned and the mesenteric defects were either closed with running, non-absorbable sutures or with non-absorbable staples (EndoHernia^™^, Medtronic, Minneapolis, MN), depending on the surgeon’s preference.

### End Points and Statistical Analysis

Statistical analysis was performed with IBM SPSS Statistics, version 23 (SPSS, Chicago, IL). Univariate logistic regression was used to investigate predictors of IH during reoperation and postoperative pain relief. Crosstabs were used to calculate sensitivity and specificity of the interpretation of the CT scan. We have used LOWESS local regression to determine the point on which %TWL could be a predictor for pain relief. Multivariate logistic regression was performed to determine independent predictors for pain relief. A *p* value < 0.05 was considered statistically significant.

## Results

A total of 193 reoperations were performed for (suspected) IH, with an estimated incidence of 2.8% (a total of 6896 LRYGB were performed in our institutions during the study period). Mean age of patients at reoperation was 41.5 ± 9.6 years and 171 (88.6%) patients were female. The median interval between gastric bypass and reoperation was 18.3 ± 19.0 months. Laparoscopic cholecystectomy was performed between LRYGB and reoperation for suspected IH in 16.0% of the study cohort (Table 1). In 35.2% of the patients, gastroscopy was performed before reoperation for suspected IH. In 28 (14.5%) patients, gastroscopy was performed after reoperation; in 4 cases, a marginal ulcer was found.

**Table 1:** Baseline characteristics

Variable	Number/mean	Range/percentage
Demographics		
Age at reoperation (years)	41.5 ± 9.6	21–61
Female gender	171	88.6%
BMI before gastric bypass^1^	42.4 ± 5.4	26–63
BMI at reoperation^1^	28.9 ± 5.7	19–56
BMI loss between LRYGB and reoperation^1^	13.6 ± 5.3	0–27
%TWL	31.4 ± 11.4	− 9.9–54.4
Interval LRYGB and reoperation (months), median	18.3 ± 19.0	0–99
Presentation and imaging		
Acute presentation	72	37.3%
CT abdomen performed	145	74.6%
CT normal	87	60.4%
Internal herniation on CT	58	40.2%
Days between CT and reoperation, median	9.5 ± 97.1	0–535
Days between IH on CT and reoperation, median	1.0 ± 21.4	0–135
Medical history		
PPI use	124	64.2%
Smoking	52	26.9%
Previous cholecystectomy		
No	114	59.1%
Before LRYGB	21	10.9%
After LRYGB but before reoperation	31	16.1%
During reoperation	19	9.8%
After reoperation	8	4.1%
Gastroscopy		
No	97	50.3%
Before reoperation	68	35.2%
After reoperation	21	10.9%
Before and after reoperation	7	3.6%

%TWL = percentage total weight loss

*BMI*, body mass index; *LRYGB*, laparoscopic Roux-en-Y gastric bypass; *CT*, computed tomography; *IH*, internal herniation; *PPI*, proton-pump inhibitor

^1^In kg/m^2^

Of all procedures, 72 (37.3%) were performed in an acute setting. Preoperative abdominal CT was performed in 144 patients, and in 56 patients (38.9%), signs of internal herniation were found. The sensitivity of the swirl sign found on CT for suspected IH after interpretation of a radiologist and an experienced bariatric surgeon was 50.0%, and the specificity was 83.0%.

An IH was found intraoperatively in 118 (61.1%) patients; there was no preference for the JJ-stomy or Peterson’s space (Table 2). There were no cases of intestinal ischemia. In 75 (38.9%) patients, no abnormalities were found intraoperatively. Nevertheless, mesenteric defects were closed in these patients in order to prevent future IH—absorbable sutures were used more frequently than staples to close the mesenteric defects (164 vs. 22). In the remaining 7 patients, absorbable sutures were used. A total of 37 patients underwent reoperation for recurrence of the postprandial, upper abdominal complaints. Four of them had recurrence of IH after previous closure with absorbable sutures, 14 had previous closure with non-absorbable sutures, and 1 had a recurrence after closure with staples. In 18 patients with symptom recurrence, there was no perioperative sign of IH. Three of them had a marginal ulcer during gastroscopy. Complete postoperative symptom relief was observed in 146 patients (77.2%). For patients in which IH was present during surgery, 82.8% had relief of pain postoperatively, as compared to 68.5% for those procedures in which no IH was found.

**Table 2:** Operative characteristics

Variable	Number (*n* = 193)	%
Conversion/laparotomy	6	3.1
Internal herniation during surgery	118	61.1
Petersen’s space hernia	54	45.8
JJ-stomy hernia	55	46.6
Peterson’s and JJ-stomy	9	7.6
Closure technique^1^		
Non-absorbable suture	164	85.0
Staples	22	11.4
Postoperative symptoms		
Relief of symptoms^2^	146	77.2
Additional reoperations		
For suspected recurrence IH	37	19.2
Proven recurrence IH	19	9.8

*JJ-stomy*, jejunojejunostomy; *IH*, internal herniation

^1^In seven patients, absorbable sutures were used

^2^Missing data of 4 patients, so total population to answer this question is 190 patients

### Predictors of Internal Herniation During Reoperation and Relief of Symptoms

There was no significant difference in the presence of IH between females and males (*p* = 0.107) (Table 3). When internal herniation was visible on CT, IH was found present perioperative in 82.8% of procedures (OR 4.78; 95%CI 2.09–10.93). In patients with normal abdominal CT, internal herniation was found in 47.3% of procedures (OR 0.20; 95%CI 0.09–0.45). In acute surgery, perioperative IH was seen more frequently than in elective surgery (OR 3.74, 95%CI 1.91–7.31).

**Table 3:** Predictors of intraoperative presence of internal herniation

Variable	Internal herniation (%)	OR	95%CI	*p* value
Age > 45 (*n* = 75)	46 (61.3%)	1.01	0.56–1.84	0.965
Female gender (*n* = 171)	101 (59.1%)	0.42	0.15–1.20	0.107
Male gender (*n* = 22)	17 (77.3%)	2.36	0.83–6.69	0.107
Reoperation after 2014 (*n* = 137)	87 (64.0%)	1.49	0.80–2.79	0.214
BMI ≥ 30 at reoperation (*n* = 73)	47 (65.3%)	1.34	0.73–2.46	0.340
BMI loss ≥ 20 (*n* = 25)	15 (60.0%)	1.04	0.45–2.43	0.926
%TWL > 40 (*n* = 44)	30 (68.2%)	1.54	0.75–3.14	0.753
Acute surgery (*n* = 73)	57 (79.2%)	3.74	1.91–7.31	< 0.001*
Elective surgery (*n* = 121)	61 (50.4%)	0.27	0.14–0.52	< 0.001*
CT normal (*n* = 87)	43 (47.3%)	0.20	0.09–0.45	< 0.001*
Internal herniation on CT (*n* = 58)	48 (82.8%)	4.91	2.11–10.93	< 0.001*

%TWL = percentage total weight loss

*BMI*, body mass index; *GB*, gastric bypass; *CT*, computed tomography

*Significant difference

A predictive factor for pain relief after delayed closure of mesenteric defects was IH on CT (OR 4.24, 95%CI 1.63–11.05). Presence of IH perioperatively affected postoperative pain relief (OR 2.21, 95%CI 1.11–4.40) (Table 4). In multivariate analysis, a positive CT scan for IH was the only independent predictor for pain relief. Time from initial LRYGB to reoperation, the location of IH, and the closure technique did not seem to affect postoperative pain relief. There was no significant correlation between smoking status and postoperative pain relief (Table 4).

**Table 4:** Predictors of symptom relief after reoperation

Variable	Symptom relief (%)	OR	95%CI	*p* value
Age > 45 (*n* = 73)	56 (76.7%)	0.95	0.47–1.91	0.889
Female gender (*n* = 167)	127 (76.0%)	0.50	0.14–1.78	0.286
Male gender (*n* = 22)	19 (86.4%)	2.00	0.56–7.09	0.286
%TWL > 40 (*n* = 44)	34 (77.0%)	1.02	0.46–2.28	0.962
Acute surgery (*n* = 70)	57 (81.4%)	1.48	0.71–3.07	0.295
Elective surgery (*n* = 119)	89 (74.8%)	0.68	0.33–1.41	0.295
Smoking (*n* = 50)	41 (82.0%)	1.48	0.65–3.34	0.352
CT normal (*n* = 86)	57 (66.3%)	0.24	0.09–0.61	0.003*
Internal herniation on CT (*n* = 56)	50 (89.3%)	4.24	1.63–11.05	0.003*
Internal herniation during surgery (*n* = 116)	96 (82.8%)	2.21	1.11–4.40	0.024*
Petersen’s space (*n* = 53)	42 (79.2%)	1.18	0.54–2.55	0.683
JJ-stomy (*n* = 54)	45 (83.3%)	1.68	0.75–3.80	0.210
Petersen’s space and JJ-stomy^1^ (*n* = 9)	9 (100%)			0.095
Closing technique				
Non-absorbable suture (*n* = 160)	123 (76.9%)	0.78	0.28–2.22	0.645
Staples (*n* = 22)	17 (77.3%)	1.00	0.35–2.89	0.998

%TWL = percentage total weight loss

*BMI*, body mass index; *GB*, gastric bypass; *CT*, computed tomography; *JJ-stomy*, jejunojejunostomy

*Significant difference

^1^As all patients with an internal herniation at both spaces had postoperative symptom relief, odds ratio could not be calculated and therefore Pearson’s chi-square was used

## Discussion

A swirl sign on CT was predictive for both perioperative presence of IH as well as for postoperative pain relief after delayed closure of mesenteric defects. Actual visible IH during laparoscopy appeared more common in an acute setting than when surgery was performed electively. Perioperative presence of IH was a predictive factor for pain relief postoperative; however, the location of the IH did not seem to affect postoperative pain relief.

The number of relaparoscopies and performed CT scans for suspected IH increased considerably over the years in our clinics. A possible explanation for this trend may be increased knowledge and awareness regarding long-term complications of LRYGB. The median interval between LRYGB and reoperation for suspected IH in our study was comparable to other studies [5, 12, 13]. Previous studies reported rapid excess weight loss (EWL) as a predictive factor for the incidence of IH, in which the risk of developing IH was twice as high in patients with rapid EWL [19, 20]. We have used LOWESS local regression to determine the point on which %TWL could be a predictor for pain relief, which was 40%. In our study, %TWL ≥ 40% as compared to a %TWL < 40% did not seem to affect the intraoperative presence of IH or of postoperative pain relief. As our study was retrospective, we did not have the exact weight loss per time period whether there was rapid weight loss could not be determined.

The presence of a swirl sign on CT was a predictor for both intraoperative presence of IH and postoperative symptom relief. However, a varying sensitivity of CT scans for diagnosing IH has been described in literature, ranging between 61 and 83% [14, 16]. The existence of intermitting IH could be an explanation for these low sensitivity rates, as the CT scans were not always performed at the moment when a patient experiences pain.

Notably, in some patients with a proven IH on CT, there was a long interval between CT and reoperation. In three patients, CT was performed in a non-bariatric hospital, where they did not acknowledge the pain as IH. Once the patients arrived in one of our institutions, the IH was seen on CT and reoperation was performed soon afterwards. Also, in two patients, symptoms had dissolved at the time of interpretation of the CT and therefore underwent elective surgery. However, we would advise to perform a reoperation when IH is seen on CT as soon as possible to reduce symptoms and prevent potential incarceration.

**Fig. 3:**
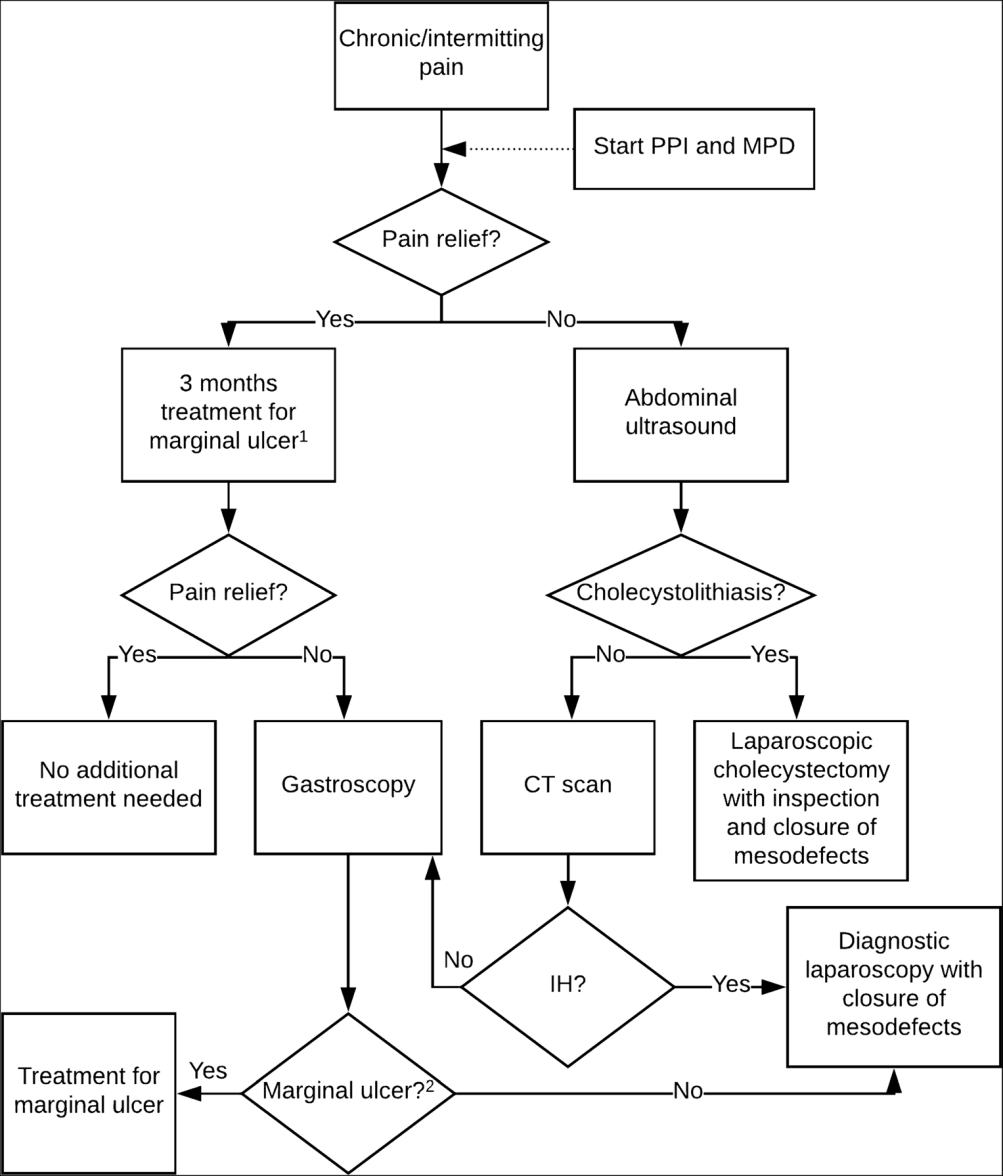
Treatment algorithm for patients with chronic and/or intermittent postprandial upper abdominal pain. (1) Treatment with PPI (proton-pump inhibitor) and MPD (mucosal protective drugs) for gastric irritation, gastritis, or marginal ulcer. (2) If a marginal ulcer is not found during gastroscopy and a CT scan has not been performed yet, a CT scan prior to diagnostic laparoscopy is recommended to exclude other abdominal pathologies

We recommend that in all patients with chronic and/or intermittent postprandial, upper abdominal pain, a treatment with PPI and mucosal protective drugs is started. If this does not give pain relief, the presence of cholecystolithiasis should first be excluded by ultrasound. If there are no gallstones detected or if the patient does not have a gall bladder anymore, we would advise to perform a CT scan in order to rule out IH. If there is no swirl sign on CT, gastroscopy should be performed to exclude the presence of a marginal ulcer. If the gastroscopy is negative as well and symptoms persist, we would advise to perform a diagnostic laparoscopy to close the mesenteric defects. Our recommended treatment algorithm for chronic and/or intermittent complaints can be found in Fig. 3.

Overall, IH was present during surgery in only 61.3% of procedures. Surprisingly, 77.2% of all patients did report postoperative pain relief after closure of mesenteric defects. In 68.5% of all procedures in which no IH was found perioperatively, postoperative pain relief was reported. Possible explanations for this observation are the intermittent presence of IH or a placebo effect of reoperation.

In our institutions, mesenteric defects were not routinely closed at primary LRYGB. Complications caused by closure of the mesenteric defects such as kinking and adhesions have been reported; however, the incidence of these complications seemed low [6, 22]. Especially after Stenberg and colleagues demonstrated the benefits of closure of the defects in their randomised controlled trial, we have decided to routinely close the mesenteric defects at primary LRYGB to reduce the incidence of IH as of January 2017 [21]. In our study, the incidence of IH is relatively low (2.8%). However, as there is no Dutch database of LRYGB of our study period, it is possible that some patients underwent reoperation for (suspected) IH in other institutions. On the contrary, we have performed reoperation in four patients who did not undergo their LRYGB in one of our institutions. Therefore, our incidence rate is an estimation. Other studies have also shown a lower incidence of IH after closure of the mesenteric defects both with non-absorbable sutures and with staples [23, 24]. However, it has also been reported that even if the mesenteric defects are closed during LRYGB, mesenteric defects might reoccur if patients have excessive weight loss [25]. In the present study, there were 37 patients with a reoperation after closure of the defects. There may have been limited experience with the closing technique of the mesenteric defects when the first relaparoscopies for suspected IH were performed. A learning curve for LRYGB of 100 procedures has been described [26, 27]. To our knowledge, there are no studies describing the learning curve for the closure of mesenteric defects. We noticed that when operating these patients, it might be easier to unravel the herniation by starting counting back from the terminal ileum. In the present study, we could not demonstrate a significant association between year of reoperation and postoperative pain relief; therefore, we expect that the learning curve for the closure of the mesenteric defects may be short.

Closure of mesenteric defects with sutures or with staples during initial LRYGB appears to result in lower incidence of IH as compared to no closure [6, 20, 21]. In the present study, there is no significant difference in the odds of symptom relief after closure of mesenteric defects with sutures as compared to staples. A limitation of this study is the small number of patients in whom staples were used to close the mesenteric defects. Further research to the difference in the use of staples versus non-absorbable sutures is recommended.

In conclusion, pain relief after closure of the mesenteric defects for (suspected) IH remains unpredictable. A swirl sign on CT was the only significant predictor of pain relief after reoperation for (suspected) IH after delayed closure of mesenteric defects of LRYGB. However, many patients benefit from closure of the mesenteric defects, irrespective of perioperative presence of IH, and therefore, reoperation for suspected IH is recommended if no marginal ulcer was found during gastroscopy.
